# The age and growth information of a ctenoid scale fossil from the Upper Cretaceous Nenjiang Formation in Songliao Basin, China

**DOI:** 10.1371/journal.pone.0303198

**Published:** 2024-05-03

**Authors:** Zhaoqing Liu, Xiaobo Li, Robert R. Reisz

**Affiliations:** 1 College of Earth Sciences, Dinosaur Evolution Research Center, International Center of Future Science, Jilin University, Changchun, Jilin Province, China; 2 Bioarchaeology Laboratory of Jilin University, Changchun, China; 3 Department of Biology, University of Toronto Mississauga, Mississauga, Ontario, Canada; Birbal Sahni Institute of Palaeosciences: Birbal Sahni Institute of Palaeobotany, INDIA

## Abstract

The study of morphological characteristics and growth information in fish scales is a crucial component of modern fishery biological research, while it has been less studied in fossil materials. This paper presents a detailed morphological description and growth analysis of a fossil ctenoid scale obtained from the Upper Cretaceous Campanian lacustrine deposits in northeastern China. The morphological features of this fossil scale are well-preserved and consistent with the structures found in ctenoid scales of extant fish species and display prominent ring ornamentation radiating outward from the central focus, with grooves intersecting the rings. A comparative analysis of the morphological characteristics between the fossil ctenoid scale and those well-studied extant fish Mugilidae allows us to explore the applicability of modern fishery biological research methods to the field of fossil scales. The scale length, scale width, the vertical distance from the focus to the apex of the scale, and the total number of radii have been measured. The age of the fish that possessed this ctenoid scale has been estimated by carefully counting the annuli, suggesting an age equal to or more than seven years. The distribution of growth rings on the scale potentially reflects the warm paleoclimatic condition and fish-friendly paleoenvironment prevalent during that period. This paper, moreover, serves as a notable application of fishery biological methods in the examination of fossil materials.

## Introduction

The morphological and microscopic features of fish scales are significant for understanding the ontogenesis and ecology of fishes [1–4]. In fishery biology, a wealth of information regarding the taxonomy, habitat, and movement patterns of fish could be obtained from fish scales [5–11]. Furthermore, in the realm of paleoichthyology, fossil scales can also be used in studies of the evolution and ontogenesis of fishes [3,12].

Modern fishery researches have underscored the utility of morphological features of fish scales in the reliable identification of genera [5–11,13]. In the survey of cycloid and ctenoid scales of Mugilidae, Zubia et al [9] found that the length scale (TLS) and width scale (WDS) features, the number of ctenii arranged in horizontal rows (HRS) and vertical rows (VRS), the total number of radius (RDS) and the vertical distance from the focus to the outer posterior margin of the scale (RS) may be critical characteristics in determining the correct systematic position of the fish. These studies have quantified pertinent parameters related to fish scale morphology, demonstrating their efficacy in elucidating information about fish life and growth patterns. Furthermore, the annular pattern on the scales’ surface provides information about individual growth and fish age [14]. The density and distribution of circulus can serve as age indicators for fish [14–17]. Nevertheless, fish scale studies are expected in fish ecology and fisheries research [4,6–11,13,18], but seldom in paleontological investigations.

In this study, a remarkably well-preserved fossil scale was recovered from dark grayish shale within the Upper Cretaceous Nenjiang Formation in the Songliao Basin, China. Growth history analysis methods are utilized to analyze the fossil ctenoid scale, encompassing calculations and the examination of its morphological and growth characteristics. Furthermore, we explore the suitability of modern fishery biological methods for studying fossil scales.

## Materials and methods

### Fossil locality and horizon

The fossil specimen in the current study was recovered from an artificial outcrop on a hill near the bank of the Songhua River (Coordinates: N44°56′46.09″; E125°03′45.49″), at Wangfu area of Songyuan City, Jilin Province, China. This site is located within the subsiding central of Songliao Basin, which is a substantial terrestrial hydrocarbon basin that developed in the late Mesozoic [19,20]. During the early and middle Late Cretaceous, the Songliao Basin experienced its peak subsidence, resulting in the expansion of the lake and continuous deposition of lacustrine sediments [19–21]. Numerous fossils, including fragmented fish and tetrapod bones, have been discovered at this location. Nevertheless, our research focuses on an exceptionally well-preserved fish scale. This fossil was excavated from the Nenjiang Formation. The geological age of this formation ranges from the Santonian to Campanian stages (79.1Ma-84.5 Ma) [19]. Fish fossils found from the Nenjiang Formation encompass chondrichthyans (Selachii), Holostei, and various teleost fishes [20,22,23].

### Methods

The fossil was fully exposed on the surface of the shale rock, which facilitated our study. To observe and verify the morphological structure and growth characteristics of the fossil ctenoid scale, we initially captured photographs in the laboratory by using a Leica DV6 microscope that equipped with ultra-deep-field function. We measured and calculated various parameters of the fossil, including TLS = scale length; WDS = scale width; RDS = the number of scale radius; RS = vertical distance from the focus to the trailing edge of the balance. We imported the resulting photographs into CorelDRAW 2019 software, which generated initial coordinates for each parameter of the scale fossil. Then, Image J 1.52a software was employed to measure and record the desired parameter length. We conducted five measurements and averaged them to obtain the final parameter length. Utilizing the photographs and measured data, we determined the placement of this fossil scale within the fish scale classification. The growth age of the scale was ultimately estimated by analyzing the distribution of annular ornamentation on its surface.

### The fundamental features of the ctenoid scale

Based on scale morphology, Agassiz [24] classified fossil and extant fish scales into four categories: cycloid, ctenoid, ganoid, and placoid scales. Bertin [25] classified cycloid and ctenoid scales as elasmoid scales. Elasmoid scale have been identified in certain basal Sarcopterygians and the majority of Actinopterygians [26,27]. Schultz [12,28] posits that elasmoid scales have their origins in ganoid or cosmoid scales, differentiating them into two primary types: amioid scale with radial ridges on the surface of the scale, and round scale with concentric rings running parallel to the edge (including ctenoid and cycloid scales). Roberts [29] further classified ’spined’ scales into three types: (1) Crenate scale (simple marginal indentations and projections); (2) Spinoid scale (spines continuous with the main body of the scale); (3) Ctenoid scale (spines separate from the main body of the scale). In the case of the ctenoid scales, the arrangement of the ctenii and the shape of the posterior field in different fishes can be found in three categories: (1) Whole ctenoid scale which possessed distinct spines or ctenii, arranged marginally and sub marginally at the posterior margin of scale; (2) Peripheral ctenoid scale that have ctenii arranged in only one row at the posterior margin of scale; (3) Transforming ctenoid scale that the ctenii are arranged in two or three marginally alternate rows and transformed into truncate submarginal ctenii. The configuration and arrangement of surface structures on scales, including circuli, radii, and ctenii, are pivotal characteristics in the study of fish taxonomy [1,2,4,5].

**Fig 1:**
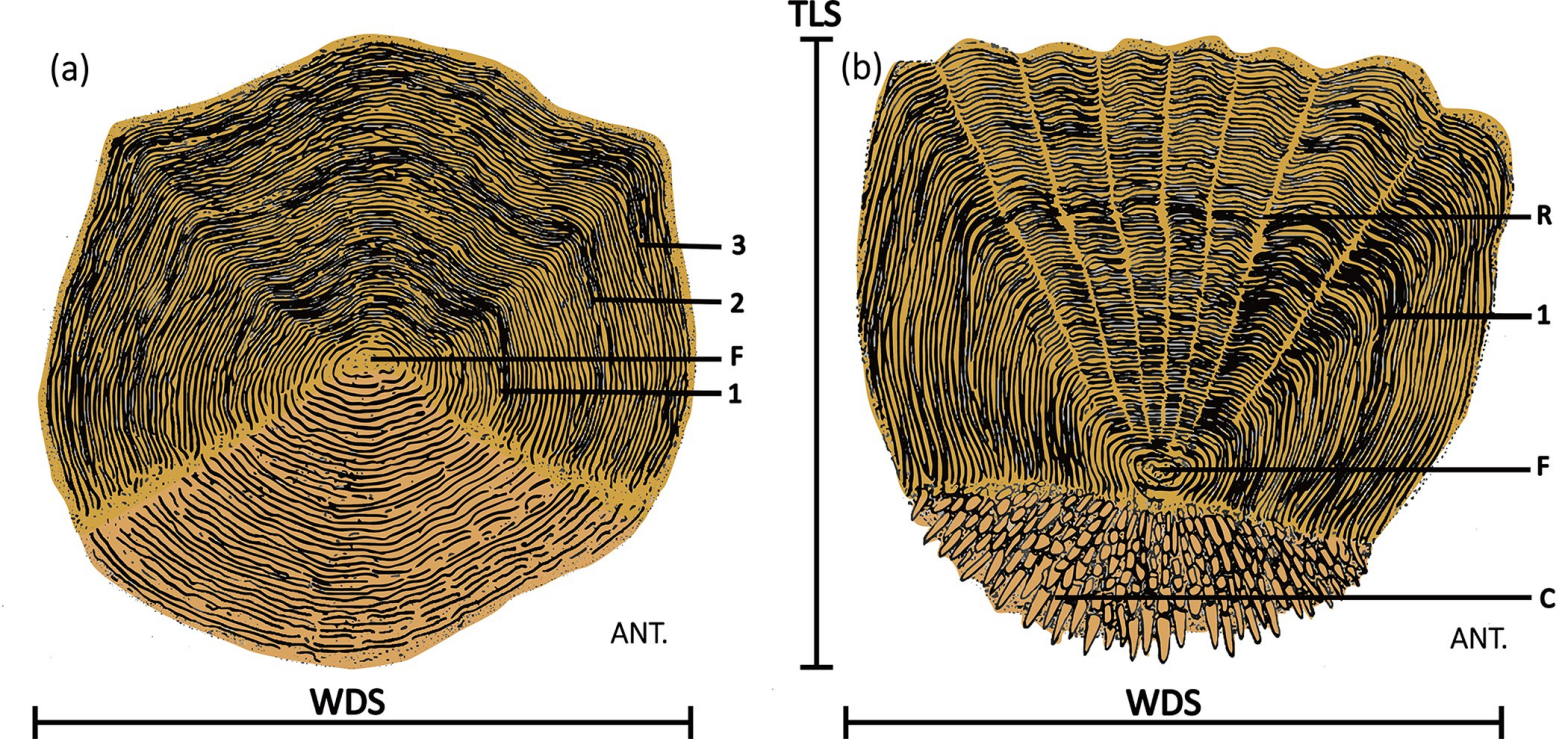
A sketch of the structural features of elasmoid scales. (a) cycloid scale; (b) ctenoid scale. Modified following Hile, 1936 [17]. Image-related explanation: Ant = Anterior; C = ctenii; F = focus; R = radii; TLS = scale length; WDS = scale width;1–3 = first to third annuli.

In ctenoid scales(Fig 1), the "focus" refers to the first part of the scale to appear in growth, often central; the"radii" are grooves that radiate from the focus to the scale margins; the "circuli" represent raised marks on the surface, usually appearing as lines which more or less follow the contour of the scale; the"annuli" markings on the surface of the scale shown to coincide with years of growth for many fishes; and the "ctenii" are tooth-like structures on the posterior part of ctenoid scales[1,2,5,17,30]. Detailed descriptions of the morphological characteristics of ctenoid scales in extant fish species are provided [1,2,5]. In the anterior region of the ctenoid scale, the ornamentation consists of circuli, parallel to the outline of the scale and is evenly spaced. The circuli are interrupted by narrow radial grooves, the so-called radii. In some species, there may be small denticles on the circuli. Radii emanate from the center of the scale, the focus. A thin layer of loose dermis overlies the posterior region and the epidermis folds around the margin of the posterior scale. The posterior area has a rounded margin and is ornamented by numerous spine-like structures, the ctenial spines, arranged in rows that make up the ctenii. There are many small tubercles in the free field of the scale, which are the residual traces of the ctenii after absorption.

In modern fishery biology, geometric morphometric methods are employed on ctenoid scales for identifying fish genera and species (e.g., Mugilidae, rattail fish) [6–11,13,18]. Morphological parameters could be used as valuable alternative tools in observing the systematic relationship between different genera or species or geographical variants [9,10]. We used parameters of extant Mugilidae ctenoid scales as a reference for this study to investigate the application of modern fisheries biology research methods in the field of palaeoichthyology. Therefore, considering the morphological and structural characteristics of the fossil material, we selected four parameters (TLS, WDS, RS, and RDS) for the comparative analysis of ctenoid scales in extant Mugilidae and the fossil scale.

**Fig 2:**
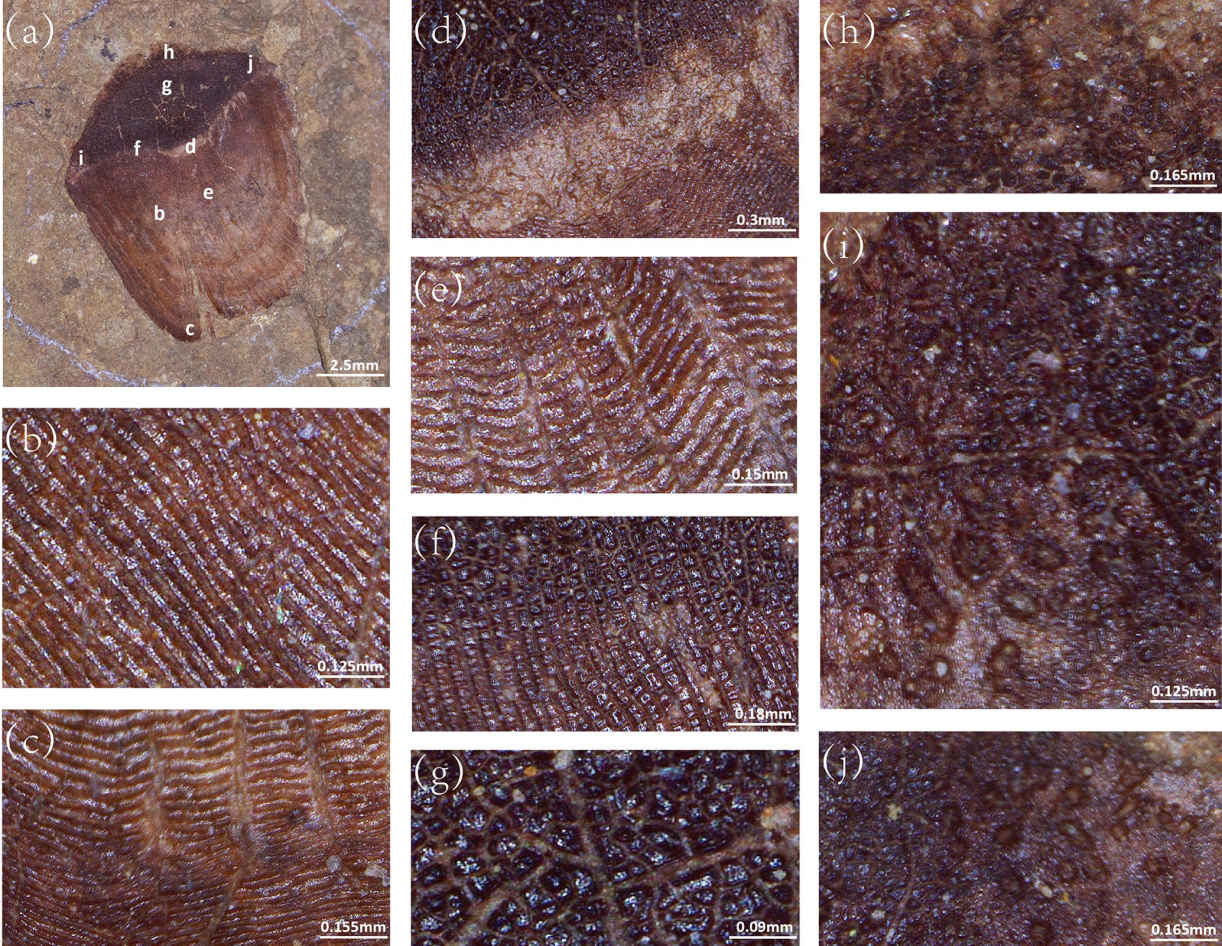
Ctenoid scale (DERC-200701-01) under Ultra-Depth Microscopy. (a) The overall picture of the ctenoid scale,(b) circulus of the sparse interval, (c) dense belt and sparse belt on the scale,(d) focus is shown, and there are no circuli in the immediate area, (e) Multiple radius from focus to end of the scale, (f) the boundary between the light brown and dark brown areas of scale, (g) posterior scale,(h) remaining brown markings on the posterior field margins, (i) remaining brown granular markings on both sides of posterior margin of scale.

The fossil ctenoid scale described in this study has essentially the same morphological structure as seen in extant ctenoid scales (Fig 2). The scale is sub-elliptic in shape. The overall color of the fossil gradually transitions from light brown to dark brown from the anterior side to the posterior side (Fig 2A). Distinct annular ornamentation can be observed in the area from the focus to the anterior side (Fig 2B). The circuli are continued from the focus to the anterior edge. Some circuli will extend to the free field. The circuli run parallel to the contour of the scale. It exhibiting a clear cycle of spaced sizes giving the ring distribution a light-dark interlacing appearance (Fig 2C). The focus (Fig 2D) of the fossil ctenoid scale is in the area near the posterior side. Grooves beginning at the focus divide these rings and run to the edge of the scale (Fig 2E). The light and dark imbricated bands of rings are trace annuli of fish scale, the grooves correspond to the radius, and both match the structure of the ctenoid scale in extant fish. A distinct separating band at the focus separates the anterior side from the posterior side (Fig 2F). The circuli completely vanish on the posterior field. There are many small black tubercles in the free field (Fig 2G). The scale is poorly preserved on the posterior margin and the ctenii structure is hard to observe. However, faint residual brown traces are visible on the posterior margin of the scale (Fig 2H). Residual brown granular marks were also found on both sides of the posterior margin of the scale (Fig 2I and 2J). There is a distinct separation of this area from the posterior field. Based on this evidence, it is likely that the region is a residual ctenii trace. The fossil scale is presumed to be a ctenoid scale according to Roberts’ [29] classification. Further classification is difficult because the arrangement of ctenii is unclear.

## Growth analysis and age estimation

Fish scales have found extensive utility in fisheries biology for purposes such as age determination, fish species identification, etc [6–11,13,18]. Generally, fish age and growth history can be determined through three methods: observing the growth of known-age fish, studying fish growth rate in relation to body size, and examining seasonal growth rings in hard tissues [14,15]. Scale growth initiates at the center (focus) and extends outward, with the ring of growth gradually increasing as the growth proceeds [14]. Although the majority of fish conform to this growth pattern, exceptions arise (e.g. *Dentex tumifrons* and yellow bream) because fish growth ring formation is impacted by both seasonal water temperature fluctuations and internal physiological adjustments stemming from cyclical changes in the external environment [15]. Fish growth characteristics are primarily influenced by nutrient availability: In summer, with ample nutrients, fish experience rapid growth, but their growth diminishes or halts in winter due to nutrient depletion [14,16,17,31]. Fish experience rapid growth during spring and summer, resulting in the formation of numerous, sparse concentric rings on their scales. This region is known as the “sparse belt” or “summer ring”. Fish growth decelerates or halts in autumn and winter, causing the spacing between scales to decrease significantly. This area is termed the "dense belt" or "winter rings." Fish age can be determined by counting the number of annuli. However, there may be other rings, such as addition rings (dummy rings) and reproductive rings {15–17, 31}. These additional rings can also impact fish age estimates. Therefore, accurately identifying these additional rings is crucial when determining age. The term ’Annuli’ is consistently used in this text to denote the annual markers for age determination.

**Fig 3:**
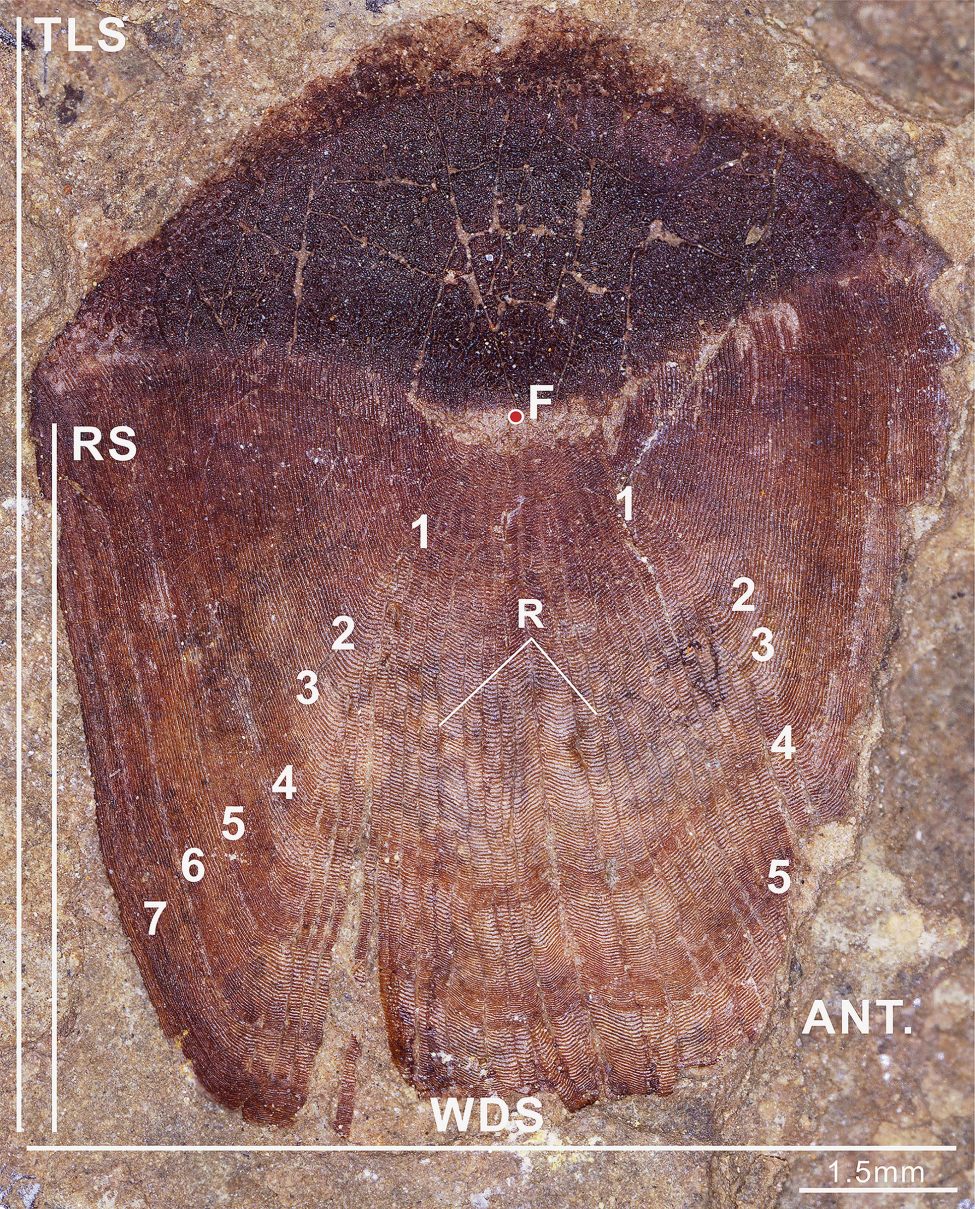
Ctenoid scale (DERC-200701-01) from the Upper Cretaceous Nenjiang Formation in Songliao Basin, China. Image-related explanation: Ant = Anterior; F = Focus; R = Radii; TLS = Scale length; WDS = Scale width; Rs = Vertical distance between focus to the apex of scale;1–7 = The annuli of the scale.

Seven annuli tracks were observed on the fossil ctenoid scale (Fig 3). The estimated age of the fish is seven years or older. As fish age, body growth slows, and the circuli become closely spaced, making it increasingly challenging to identify annual markers [14,16,17]. This can lead to significant errors in determining the age of older fish. Consequently, it is highly likely that the fossil scale is older than seven years. This age is common among modern freshwater fish and typically indicates a mature developmental stage [9–11]. On the other hand, the distal end of the fossil scale displays a densely clustered pattern of distinctive rings (Figs 3 and 6I). Fish growth can be categorized into three stages: the initial stage features immature fish with rapid growth; the second stage involves sexually mature fish with relatively consistent growth rates; the third stage is senescence, marked by a metabolic decline and slowed growth until death [15]. Due to the unique distribution pattern of fossil growth rings, it is likely that the fish was in the senescence stage. Nonetheless, the existing materials cannot determine whether the fish died naturally or due to other factors.

**Fig 4:**
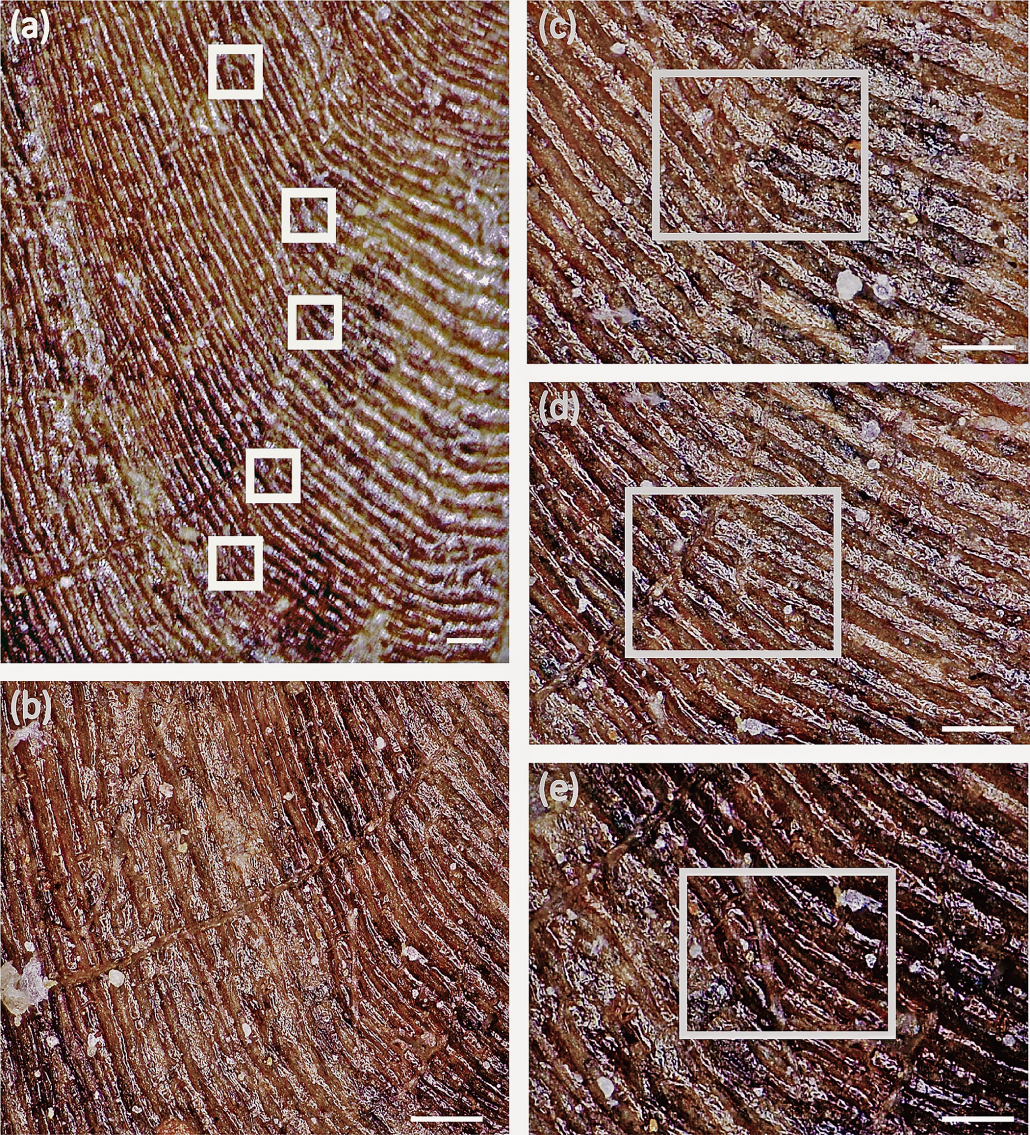
Image of ctenoid fossil growth ring under ultra-depth microscope (DERC-200701-01). The scale is 50 μm. (a) the Incomplete rings in the sixth year of scale growth; (b) the broken area between the fourth and sixth years; (c)-(d) enlarged views of the incomplete rings during the sixth year.

Indeed, relying solely on the brightness distribution to determine age can lead to significant errors since some scales do not exhibit alternating patterns of light and dark on their surface [17,31,32]. Irregularly distributed or fractured rings on fish scales can assist in annuli identification [14,16,17,31]. The final circuli in summer do not fully encircle the scale, whereas the circuli in the subsequent spring completely encircle the scale, creating a broken ring that is typically easily distinguishable [14–17,31,32]. The fossil scale exhibit distinct seasonal ring structures with alternating light and dark sections (Fig 3). Additionally, a significant number of incomplete circuli have formed on the scale (Fig 4). These dummy rings are irregularly distributed across the anterior portion of the scale, occurring in both sparse and dense areas. It appears that these dummy rings occur multiple times throughout the year. For instance, at least seven dummy rings were identified between the start and end of the sixth year (Fig 4A). Typically, incomplete ring growth occurs during periods of very slow growth of fish scales, often in the winter [17]. Changes in growth rates can also be attributed to various sources of stress, including spawning, transitions between freshwater and saltwater, parasitism, injury, favorable or adverse environmental conditions, or health conditions [14–17,31,32]. Consequently, a significant number of dummy rings signify highly intricate alterations in the fish’s growth environment and living conditions. This may indicate that the overall growth rate of the fish is slower, even during the summer when nutrition is adequate, and cannot sustain the full development of the ring. It is noteworthy that irregularly broken or densely packed circuli often develop at the termination of the dense belt on the fossil scale (Fig 4B). Consequently, these markers assist in the determination of the fossil scale’s age.

Ecologically, in warmer aquatic environments, fish scales tend to exhibit alternating light and dark patterns [15]. In the study of paleoclimate within the Nenjiang Formation, several indicators, including element ratios (Fe/Mn and Mg/Ca), the total organic matter (TOC), isotopic values (δ^13^C_carb_ and δ^18^O_carb_), and clay mineral composition of lacustrine carbonates, collectively suggested a warm paleoclimate during the late Cretaceous [33,34]. Therefore, the distinctive distribution pattern of growth rings may indeed indicate the relatively warm paleoclimate of that era. Significant variations in growth rates and patterns likely existed among fish of various genera and species. Nonetheless, insights from the fossil ctenoid scale can illuminate fascinating aspects of Cretaceous fish growth. To summarize, the key factor in estimating the age of fossil scales hinges on interpreting the annuli. Nevertheless, estimating this can be challenging due to factors like weathering, abrasion, and the closely spaced arrangement of the rim circuli. Naturally, the results may be susceptible to errors related to scale preservation quality, computational inaccuracies, and the evolutionary development of scales. Furthermore, other ring types, like reproductive rings, have not been definitively identified on this fossil. Additional information about fossil ctenoid scales requires new materials and further research.

## Discussion

### Comparison with scales of extant fish

The extent fish Mugilidae, their scale morphology has been extensively examined and employing statistical methods to analyze scale-related parameters can yield valuable insights about the scale’s owner [9–11]. In the Mugilidae ctenoid scales, the focus of the head scales is closer to the center, while in other positions, it is situated nearer to the posterior edge of the scales [9]. Comparing these characteristics with those of the fossil scale indicates that the fossil likely does not belong to the front of the fish body but rather to its lateral body. The area on the fish’s body with the least scale variation was identified below the dorsal fin and above the lateral line and analyzing scales from this region yielded more dependable results [35]. Hence, this study compares the pertinent parameters of the fossil scale with those of the transverse ctenoid scale found in extant Mugilidae.

**Fig 5:**
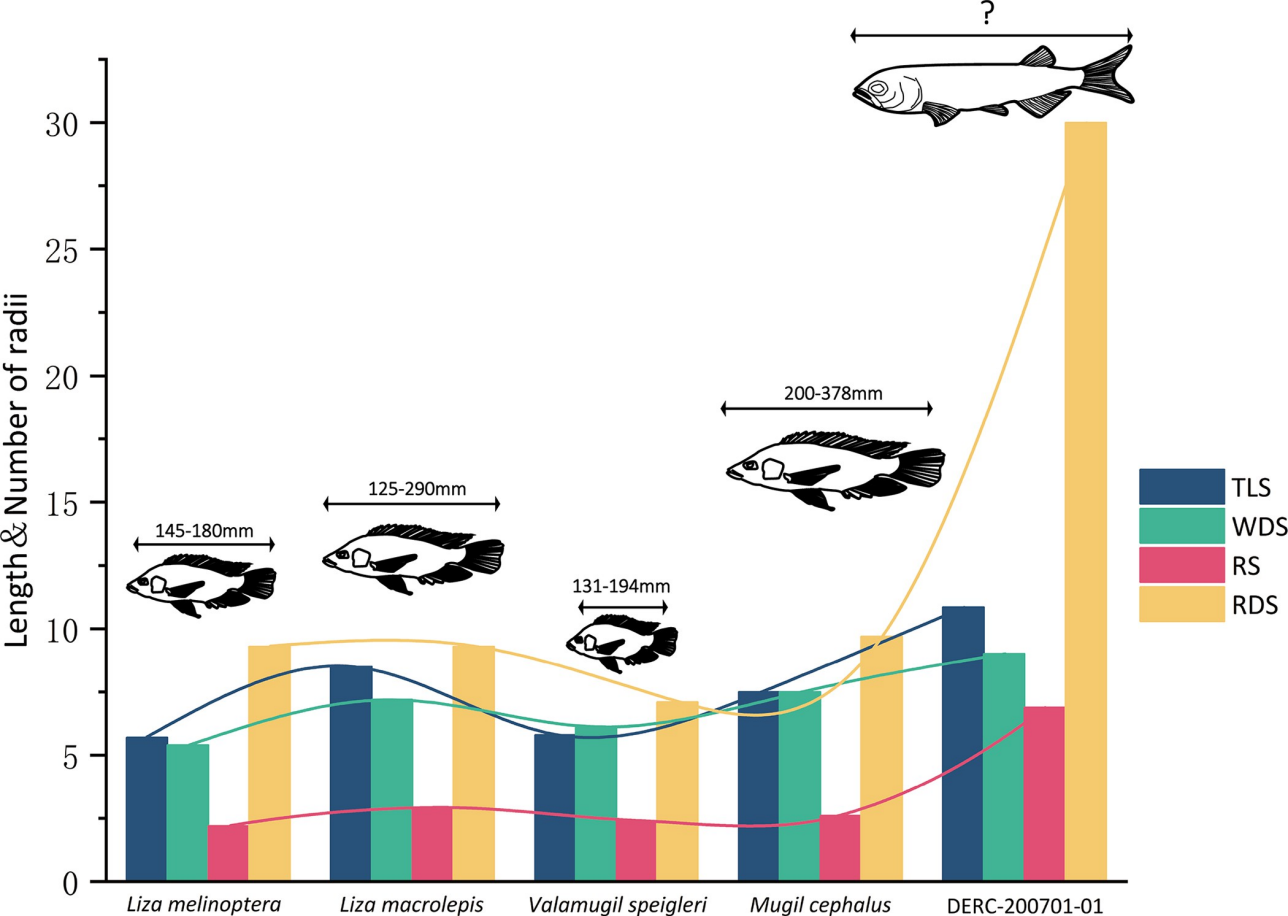
Comparison diagram of various parameters. Fossil scale TLS = 10.853 mm, WDS = 9.105 mm, RS = 6.882mm, RDS = 30. Mugilidae data from Zubia et al.,2015 [9]. The unit of length is mm.

**Fig 6:**
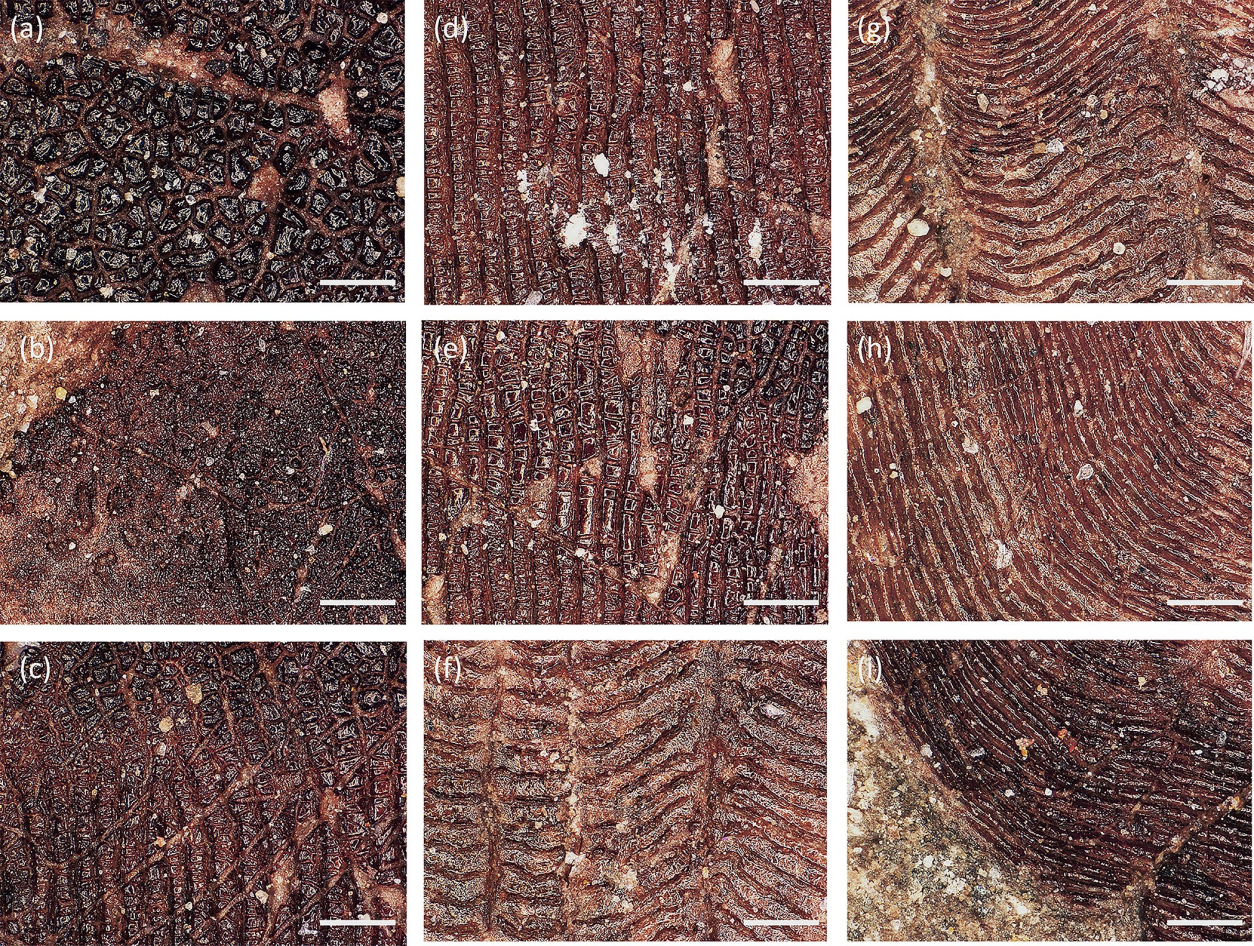
Fossil characteristics of ctenoid scales related to morphology and functions (DERC-200701-01). (a) shows small black tubercles; (b) shows large brown granular process; (c) shows the transition between the anterior field and posterior field, (d) and (e) show small particles in the anterior field; (f)-(h) shows circuli and radii near the leading edge of the scale, and there are no small particles on these circuli; (i) showcases the circlulus with very tightly arranged scales at the edges. The scale is 100 μm.

Modern ctenoid scale data from Cichlids was chosen for comparison in this study because it has undergone comprehensive and exhaustive analysis [1,6–11,13]. Consequently, we compared the four parameters (TLS, WDS, RS, and RDS) of the fossil scale with those of the extant ctenoid scales (Fig 5). Conducting a comparison led to the following conclusions: (1) The TLS, WDS, RS, and RDS values for the fossil scale are significantly larger than those for Mugilidae according to Zubia [9], suggesting that the main body of the fossil may have exceeded 400 mm in length. (2) Compared to the ctenoid scale of extant Mugilidae, the RS of the fossil scale is notably longer even when TLS and WDS are similar. The focus of the ctenoid fossil scale is closer to the posterior edge. Mir’s [18] study on the Indian carp Labeo rohita revealed that the focus of the cycloid scale tends to be toward the front or center, while the ctenoid scale’s focal point is primarily positioned at the rear edge or top. This provides additional evidence supporting the scale’s ctenoid nature. (3) Fossil ctenoid scale possess significantly more RDS than their extant counterparts. The direction of the radii aligns with the flow, and a higher number of radii suggests that the fish may have increased swimming speed and greater scale flexibility [10,36,37]. Thus, the main body of this ctenoid scale likely possessed exceptional flexibility. Moreover, research has demonstrated that the ctenii and circuli enhance the overall durability of fish scales, aiding in protection against threats and movement [38–40]. Numerous ctenii traces along the fossil scale’s periphery (Fig 6A and 6B). Prominent brown granular formations line the scale edge, accompanied by numerous small black tubercles emerging behind them. These diminutive tubercles progressively diminish from the scale’s center towards its periphery, ultimately transforming into sizable granular deposits. Similar patterns are observed in Cichlidae, signifying the absorption of ctenii [1]. Dense minute particles have also formed on the circuli, predominantly concentrated in the circuli vicinity adjacent to the focus yet absent from those near the scale’s edge (Fig 6D–6F and 6H). These tubercles in the posterior scale region and the tiny particles in the anterior region contribute to friction through mechanical anchoring, effectively preventing scale slippage [1]. These characteristics of ctenoid scales may enhance their reproductive success and predator avoidance. This could be a significant factor in explaining why ctenoid scales have persisted alongside other scale types throughout the evolutionary history of fish scales. Additionally, this could be a contributing factor to the relatively stable form and structure of ctenoid scales over the past 70 Ma.

### Cretaceous ctenoid scale: Implications for fish scale evolution

Comprehensive research on fossil ctenoid scale is warranted to explore their biological significance, considering their distinctive morphological features and valuable insights into paleoecology. Paleontological reports on ctenoid scales are exceedingly scarce. Traquair [41] reported the discovery of Middle Carboniferous *Cryphiolepis* elasmoid scales, which he labeled as "cycloidal" but provided limited scale character descriptions. Mickle [42] noted that Carboniferous *Guntherichthys* scales exhibit rounded margins. Unfortunately, the poor preservation of this fossil scale has hindered the observation of distinct features. Becker [43] discovered a scale resembling a "ctenoid scale" with multiple circuli in marine sediments at the Cretaceo-Tertiary boundary. However, it does not meet the criteria for a “true” ctenoid scale; instead, it belongs to the Crenate or Spinoid type and is poorly preserved [43]. The most recent report on fossil ctenoid scales discusses Middle Eocene *Palaeoperca proxima*, revealing annuli traces on these fossils [32].

The fossil ctenoid scale found from the Nenjiang Formation of the Songliao Basin, contributes novel insights to the Cretaceous fish fossil record of Northeast China. The Nenjiang Formation yields a diverse fossil fish, encompassing chondrichthyans (Selachii), Holostei, and teleostei (e.g., *Hama macrostoma*, *Sungarichthys*, *Jilinichthys*) [20,22,23]. While these fish fossils typically preserve intact bones, complete scales with well-defined morphological features are less common. The scales of Cretaceous fish genus *Lycoptera*, *Paralycoptera*, *Jinanichthy*, and *Kuyangichthy* discovered in northeastern China are generally considered to be cycloid scales [23]. However, some researchers have argued that there is no inherent demarcation between cycloid and ctenoid scales, except for the serrated posterior edge or the presence of ctenii in the latter [12,28,29]. Studies have demonstrated the coexistence of ctenoid and cycloid scales on the same fish, and variations in individual development may lead to the presence of different scale types on the same kind of fish [44–46]. Further evidence is needed to ascertain the presence of this scale in primitive teleostei fish with cycloid scales within the Songliao Basin. This study marks the initial discovery of a ctenoid scale in the Songliao Basin. Another important finding is that the fish fossils bearing cycloid scales found in Northeast China tend to have relatively modest body lengths, typically ranging from 10 mm to 300 mm [23]. Additionally, present-day fish possessing ctenoid scales tend to be of smaller size, including carp, Mullidae, and Mugilidae [9,10,46]. Notably, Ichthyoctiformes (*Xiphactinus*), the largest known teleost fish from the late Cretaceous in North America, includes complete specimens reaching approximately five meters in length, with isolated elements from even larger individuals [47–49]. These scales exhibit punctae in the posterior areas, in addition to radii and circuli. These fish are marine species characterized by their substantial size. These punctae likely represent remnants of ctenii. Both Ichthyoctiformes and teleostei in Songliao Basin are considered primitive taxa [23,47]. Hence, we speculate that the ctenii are likely a plesiomorphy of more primitive Osteichthyes, representing a progressive form of fish scale evolution. This fossil scale might indicate the presence of large-bodied fish in northeastern China during the Cretaceous period. However, such speculation needs to be supported by more material in this area. Additionally, the presence of this scale in the Late Cretaceous strata of China is particularly noteworthy, warranting a more comprehensive examination of fish fossils in this region. Nonetheless, the discovery of this scale provides evidence suggesting the potential evolution of fish with ctenoid scales during the Cretaceous period, and conceivably, even earlier. Further investigation of Cretaceous ctenoid fossils could aid in elucidating the intricacies of early scale evolution and development.

### Fossils illuminate potential of fishery biology

Modern fishery biological research enables the study of life history, growth rate, age of sexual maturity, ovulation, and spawning habits through the analysis of fish hard tissue imprints [15]. The morphology of the late Cretaceous fossil ctenoid scale employed in this study closely resembles that of extant fish. The measurement of the distance between the focus and annuli can be used inversely to estimate the length of fish body, providing insights into the habitat and nutrient intake [18,50]. In Mugilidae studies, a distinction was made between two morphologically similar *Mugil* (*M*. *cephalus* and *M*. *curema*) using ctenii morphology [13]. Matondo et al [51] discovered significant differences in the ctenoid scales between male and female fish in the Mullidae. Arola [52] and Sudo et al [37] examined the mechanical properties and surface morphology of elasmoid scales, investigating how fish movement patterns are reflected in scale morphology. Zubia and Ana et al have demonstrated the possibility of classifying fish genera using morphometric and statistical methods based on ctenoid scale characteristics [6–11,13]. The question is could exact morphometric measurements be employed to estimate the size of ancient fishes as has been done for extant taxa? Another question is could the life history or health status of ancient fish be elucidated through the analysis of ring distribution patterns in scales, akin to modern fishery biology practices?

Our study represents a promising avenue of research that amalgamates modern fisheries biology with paleontology. Currently, as scales are comprehensively investigated in modern fishery biology, corresponding scale fossils have the potential to reveal extensive insights into the life patterns and ecological surroundings of ancient fish. It appears that acquiring the necessary details to preserve intact scale fossils has become the most formidable obstacle. All of these speculations necessitate larger quantities of fossil material and more comprehensive studies to ultimately ascertain the value and significance of employing modern fishery biology methods in fossil scale research.

In this study, we analyzed fossil ctenoid scale by using modern fishery biological methods to reveal crucial information about their age, life history, and morphological function. Our primary focus was on revealing the valuable information concealed within these isolated scale fossils, rather than using them exclusively for phylogenetic analysis. The information within these scales can provide insights into particular paleoecological aspects of the lake environment. Furthermore, this study confirms that traditional age estimation methods for scales apply to Cretaceous paleo-fish scales, despite the inherent uncertainties and variability associated with our findings. The discovery of this ctenoid scale fossil in the Upper Cretaceous strata of Songliao Basin enriches the fossil fish record of northeast China and provides evidence for the early presence of fish with well developed ctenoid scales. Furthermore, the outcomes of this research bolster the utilization of modern fishery biological methods on fossil fish scales.

## Conclusion

1. The fossil fish scale from the Upper Cretaceous display three morphological features on their surfaces: focus, radii, and circuli, which closely resemble those found on extant fish ctenoid scales. Additionally, there is evidence of ctenii structures on the posterior edge of the scute. These characteristics definitively confirm the accurate classification of the fossil scale as a ctenoid scale, offering substantial evidence for the existence of well developed ctenoid scales as far back as the Late Cretaceous.
2. The TLS, WDS, RS, and RDS values of the fossil ctenoid scale all surpass those of the extant ctenoid scale chosen for comparison within Mugilidae. Fish from the Late Cretaceous period that possessed ctenoid scales might have exhibited large body size and increased physical flexibility, potentially contributing to their enhanced survival capabilities. This may be one of the reasons why the ctenoid scale has persisted and evolved into the predominant type of scale on the body surface of fishes over the course of their long evolutionary history.
3. The fossil ctenoid scale exhibits 7 annuli on its surface, suggesting that the age of the fish is estimated to be at least 7 years. We speculate that the fish had been in a growth stage and living conditions were good with adequate availability of nutrients before death. Furthermore, the distinctive distribution pattern of the rings could also indicate a warm Late Cretaceous period in northeast China.
4. This research underscore the feasibility and potential of applying modern fishery biological research techniques to the study of ancient fossil fishes.

## Supporting information

S1 File (PDF)
