# Supplementary material for: The age and growth information of a ctenoid scale fossil from the Upper Cretaceous Nenjiang Formation in Songliao Basin, China
Source: PLoS One. 2024 May 3;19(5):e0303198. doi: 10.1371/journal.pone.0303198 (PMC11068172; doi:10.1371/journal.pone.0303198)
Supplement: S1 File — (PDF) [file pone.0303198.s001.pdf]

Measurement of Fig.5

	TLS/mm	WDS/mm	RS/mm	RDS
Liza melinoptera	5.7	5.4	2.2	9.3
Liza macrolepis	8.5	7.2	2.9	9.3
Valamugil speigleri	5.8	6.1	2.4	7.1
Mugil cephalus	7.5	7.5	2.6	9.7
Fossil ctenoid scale	10.853	9.015	6.882	30

Comparison diagram of various parameters. The unit of length is mm. Fossil scale TLS=10.853 mm, WDS=9.105 mm, RS=6.882mm, RDS=30. Mugilidae data from Zubia et al.,2015[9]
